# Exploring Supernumeraries - A New Marker for Screening of B-Chromosomes Presence in the Yellow Necked Mouse *Apodemus flavicollis*

**DOI:** 10.1371/journal.pone.0160946

**Published:** 2016-08-23

**Authors:** Vanja Bugarski-Stanojević, Gorana Stamenković, Jelena Blagojević, Thomas Liehr, Nadezda Kosyakova, Marija Rajičić, Mladen Vujošević

**Affiliations:** 1 Department of Genetic Research, Institute for Biological Research “Siniša Stanković”, University of Belgrade, Belgrade, Serbia; 2 Molekulare Zytogenetik, Institut für Humangenetik und Anthropologie, Universitätsklinikum, Jena, Germany; Leibniz-Institute of Plant Genetics and Crop Plant Research (IPK), GERMANY

## Abstract

Since the density of simple sequence repeats (SSRs) may vary between different chromosomes of the same species in eukaryotic genomes, we screened SSRs of the whole genome of the yellow necked mouse, *Apodemus flavicollis*, in order to reveal SSR profiles specific for animals carrying B chromosomes. We found that the 2200 bp band was amplified by primer (CAG)_4_AC to a highly increased level in samples with B chromosomes. This quantitative difference (B-marker) between animals with (+B) and without (0B) B chromosomes was used to screen 20 populations (387 animals). The presence/absence of Bs was confirmed in 96.5% of 342 non mosaic individuals, which recommends this method for noninvasive B-presence detection. A group of 45 animals with mosaic and micro B (μB) karyotypes was considered separately and showed 55.6% of overall congruence between karyotyping and molecular screening results. Relative quantification by qPCR of two different targeted sequences from B-marker indicated that these B-specific fragments are multiplied on B chromosomes. It also confirms our assumption that different types of Bs with variable molecular composition may exist in the same individual and between individuals of this species. Our results substantiate the origin of Bs from the standard chromosomal complement. The B-marker showed 98% sequence identity with the *serine/threonine protein kinase VRK1* gene, similarly to findings reported for Bs from phylogenetically highly distant mammalian species. Evolutionarily conserved protein-coding genes found in Bs, including this one in *A*. *flavicollis*, could suggest a common evolutionary pathway.

## Introduction

An extremely heterogeneous group of supernumerary chromosomes, nonessential for organism viability, exists in approximately 15% of cytogenetically examined eukaryotic species [1] and new ones are regularly discovered. They were marked as B chromosomes [2] and the tag Bs has remained as an illustration of their proposed origin from the standard complement of autosomes and sex chromosomes (As). After more than a century of intensive research, Bs continue to be one of the most enigmatic components of the genome. In spite of their disposability and contrary to other accessory karyotype elements, Bs have the special ability of maintenance in certain species through numerous generations. They are variable in size, number, morphology, phenotypic effects and ways of transmission, have irregular modes of inheritance, pairing incapacity with As during meiosis and, being dispensable, they follow their own evolutionary path [3]. Learning about their biology could help us to understand better the evolution of genomes and gene regulation under varying amounts of selection pressure [4–6].

Bs have been reported in 75 mammalian species, mostly in rodents [7,8]. The genus *Apodemus* is a suitable mammalian model to study Bs, possessing rather uniform karyotypes, while Bs appear in one third of approximately twenty described species [9]. They occur in both main subgenera, *Apodemus* (*A*. *peninsulae*, *A*. *agrarius*) and *Sylvaemus* (*A*. *flavicollis*, *A*. *sylvaticus*, *A*. *mystacinus*), reviewed in [10,11] and additionally in the Japanese endemic species *A*. *argenteus* [12]. The occurrence of Bs is not in accordance with the phylogeny of this genus [13]. Thus, *A*. *peninsulae* and *A*. *flavicollis*, species with the highest frequency of Bs, belong to lineages that diverged 8–10 million years ago [14]. Species closely related with each of them have sporadic incidences of Bs, e.g. *A*. *agrarius* [15] and *A*. *sylvaticus* 2.4% [16], or do not carry them at all.

Again, in *A*. *peninsulae* and *A*. *flavicollis* Bs are recorded in populations over a wide distributional area. The number and morphology of Bs may vary substantially between populations, individuals, tissues and cells. In populations of *A*. *peninsulae* they were divided into four groups according to their morphology as detected by Giemsa and DAPI staining [17]. In *A*. *flavicollis* and *A*. *sylvaticus*, Bs are euchromatic and indistinguishable from the standard complement by conventional staining and they share the same G- and C-band distribution. Two types of Bs were recorded in *A*. *flavicollis*: smaller than the smallest A chromosomes [18,19] and Bs of the same size as the five smallest A chromosomes [20]. Reported B chromosome prevalence ranged from 11% to 63% in 13 out of 14 populations studied in former Yugoslavia [21,22]. A possible reason for their long-term presence in populations could be their contribution to: genetic variability, adaptive effects and population dynamics [22–25].

Numerous results confirmed that Bs of various species carry repetitive, often multiplied, DNA sequences [13,26–32]. Several classes of repetitive DNA, 5S and 45S ribosomal DNA (rDNA), satellite DNA, histone genes, small nuclear DNA, mobile elements, and organellar sequences were recorded in Bs of different species [33]. Highly repetitive DNA sequences grouped in specific heterochromatic regions of chromosomes have already proved to be a convenient marker in studies of karyotypic evolution and phylogeny reconstructions [34]. However, there are few investigations dealing with the origin and molecular structure of B chromosomes from *Apodemus* species [11,13,28,30,35]. Repetitive DNA sequences were confirmed in Bs from *A*. *peninsulae*. Two types of B-specific heterochromatin of *A*. *peninsulae*, one indicated as genus specific, were reported using FISH [28]. B chromosomes in the Korean field mouse were analyzed by FISH [13] with DNA probes generated by microdissection of As and Bs, followed by DOP-PCR and the repeats were classified in relation to their homology and predominant location. It was shown [11] that Bs and As of *A*. *peninsulae* share three types of repetitive sequences, suggested that Bs originated from fragments of As and further hypothesized that Bs occurred independently in this lineage. Detailed research on B-specific repetitive sequences in *A*. *flavicollis* would help to clarify the origin and structure of B chromosomes in this genus.

Starting from the fact that the density of SSR may vary between different chromosomes of the same species in eukaryotic genomes [36], we used the Inter Simple Sequence Repeat-Polymerase Chain Reaction (ISSR-PCR) in order to reveal distinctive SSR profiles characteristic for B carriers. This method, introduced by [37] is commonly employed in genome polymorphism and phylogenetic studies of various taxa [38–41]. Further, we aimed to analyze nucleotide sequences of the obtained ISSR distinctive DNA profiles.

ISSR screening with primer (CAG)_4_AC proved to be a simple, noninvasive and highly specific method for detection of B presence in *A*. *flavicollis*. B-marker was further mapped in the metaphase chromosomes by Fluorescent In Situ Hybridization (FISH) method. The high level of identity of the B-marker with *serine/threonine-protein kinase* (*VRK1*) gene sequences contributes to the growing list of protein-coding genes recorded in B chromosomes.

## Materials and Methods

### Samples and locations

This study included a total of 387 specimens of *A*. *flavicollis* (Table 1), collected from twenty different localities in Serbia in the period 2002–2015, using Longworth traps. The total sample included 203 males and 184 females. We also analyzed two samples of *A*. *sylvaticus*, one of them harboring Bs, in order to verify the efficiency of the method in another species. This research was conducted under permits issued by the Ministry of natural resources, mining and spatial planning, Republic of Serbia (number: 353-03-250/2010-04). The locations sampled were not privately owned or protected in any way, and this field study did not involve endangered or protected species. The animals were treated according to Directive 2010/63/EU of the European Parliament and the Council of 22 September 2010 on the protection of animals used for scientific purposes. All animal procedures were approved by the Ethical Committee for the Use of Laboratory Animals of the Institute for Biological Research ‘‘Siniša Stanković”, University of Belgrade. Following recommendation, animals were sacrificed using ether.

**Table 1:** Comparison between results of karyotyping and ISSR-PCR profiling with primer (CAG)_4_AC on a total sample of 387 individuals of *A*. *flavicollis*.

Method	0B	+B			n1	mosaics			n2
		1B	2B	3B		1-nB	0-nB	1μB	
Karyotype	262	58	16	6	**342**	9	30	6	**45**
ISSR-PCR	255	53	16	6	**342**	8	14	3	**45**
Congruence (%)	97.3	91.4	100	100	**96.5**	88.9	46.7	50	**55.6**

n1- total number of non mosaic samples; n2- total number of mosaic and μB samples.

### Chromosome analyses

Chromosome number was determined by conventional cytogenetic analysis. Chromosome preparations were obtained directly from bone marrow using standard techniques. A minimum of thirty spreads from each animal was examined to detect and determine the number of Bs.

### Extraction and purification of genomic DNA (gDNA)

Tissue material was taken from all animals and frozen at −20°C. Total DNA was extracted from the liver, heart, ear, tail and buccal swab using DNA extraction kits (DNeasy Blood and Tissue Kit, Qiagen and Genomic DNA Purification Kit, Thermo Scientific). Additional DNA extractions from several tissues (heart, ear, tail and buccal swab) from the same mouse were tested for the ability to gain B-marker. In order to ensure reproducible and reliable ISSR amplification reactions [42,43] we measured the quantity, purity and integrity of isolated gDNA by spectrophotometry and agarose gel electrophoresis.

### ISSR-PCR analysis

For discrimination of B-specific DNA profiles we tested twelve ISSR primers (Metabion, Germany) with dinucleotide and trinucleotide repeats: (CAG)_5_, (CAA)_5_, (CAG)_5_GC, (CAG)_4_AC, (CAA)_5_GC, (CAA)_4_AC, (CA)_6_CG, (CA)_8_TA, (GA)_6_GC, (GA)_8_AC, (CA)_7_ and (GA)_7_. There were eight anchored primers with a dinucleotide tail attached to the 3’ end and four non-anchored ones. PCR were optimized according to [44]. Optimized conditions for a final volume of 20 μl were: PCR reaction buffer [750 mM Tris–HCl (pH 8.8 at 25°C), 200 mM (NH_4_)_2_SO_4_, 0.1% Tween 20] (Thermo Scientific) 2.50 mM MgCl_2,_ 1mM dNTPs, 0.5*μ*M primer, 1 U of *DreamTaq* DNA Polymerase (Thermo Scientific, Waltham, MA USA) and 20 ng of gDNA. The temperature profile was: initial denaturation at 94°C for 5 min, 45 cycles (94°C for 30 s; 58°C for 30 s; 72°C for 1.5 min) and a final extension at 72°C for 7 min. We used 200 μl microtubes in a Thermal Cycler 2720 (Applied Biosystems, Boston, CA, USA). Amplification products were separated by agarose gel electrophoresis. The DNA fragments were visualized with UV light using the Bio-Rad Gel Doc XR+ System and the results photo-documented using Quantity One 1-D Analysis Software (Bio-Rad Laboratories, Inc., California, USA).

### Restriction Fragment Length Polymorphism (RFLP) Analysis

2200 bp bands amplified from gDNA of five +B and five 0B mice were removed from the agarose gels, purified with QIAquick Gel Extraction Kit (Qiagen) and then digested with twelve restriction endonucleases (RE): *Apa*I, *Hind*III, *Bsp*120I, *Mfe*I, *Hpa*I, *Msp*I, *Mbo*I, *Alu*I, *Mse*I, *Ecl*136II, *Hae*III and *Sac*I (FastDigest, Thermo Scientific, Waltham, MA USA) according to the manufacturer’s instructions. Digested DNA fragments were separated by agarose gel electrophoresis, visualized and photo-documented as above.

### Cloning and sequencing

The 2200 bp B-specific DNA band, amplified from a random 1B animal, was removed and purified for sequencing. Cloning and sequence analysis of ten different clones was done in both directions on ABI 3730XL capillary sequencers by a third party (Macrogen, Europe). The aligned sequences were compared and analyzed using softwares BioEdit ver.7.2.5 [45] and MEGA ver. 6 [46] and also in the NCBI GenBank.

### Primer design and End-Point PCR

**Fig 1:**
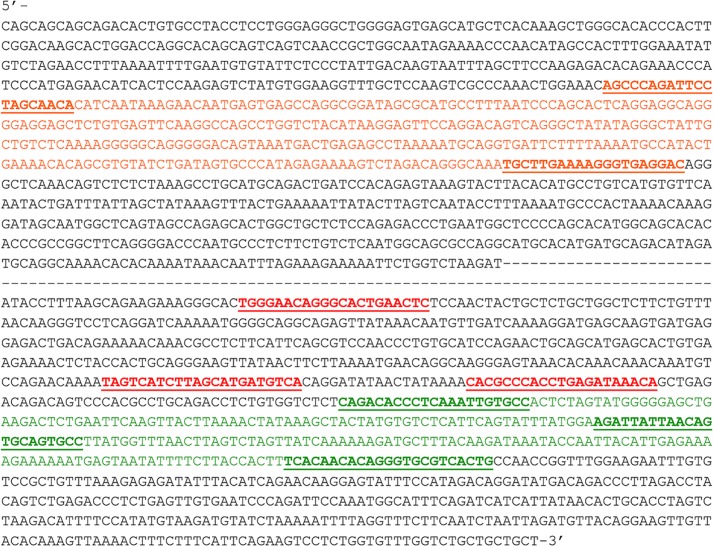
Sequence of the B-specific 2200 bp ISSR Amplicon Obtained from gDNA of a Random Animal with 1B Karyotype. Both, 991 nt at the 5’-end and 995 nt at the 3’-end listed here were completely identical among all ten clones. Approximately 200 intermediate bases without readable sequence results were omitted from the shown sequence. Sequences of eight designed specific primers are underlined: *DD5F*, *DD5R*, *SR1F*, *SR1R*, *SR2R*, *LD1F*, *LD1R*, and *LD2R*. Sequences of two fragments amplified by qPCR are colored in orange at 5’ end (321 bp) and green at 3’ end (251 bp).

To further verify the acquired B-marker by ISSR-PCR, we designed primers from different parts of the 2200 bp sequenced fragment (Fig 1), using the Primer3 ver.0.4.0 tool [47] and OligoAnalyzer 3.1 [48]. Primer sequences were as follows: *DD5F*: 5’-AGC CCA GAT TCC TAG CAA CA-3’; *DD5R*: 5’-GTC CTC ACC CTT TTC AAG CA-3’ (Invitrogen, Life Technologies, Waltham, MA USA) *SR1F*: 5’-TGG GAA CAG GGC ACT GAA CTC-3’; *SR1R*: 5’-TGA CAT CAT GCT AAG ATG ACT A-3’; *SR2R*: 5’-TGT TTA TCT CAG GTG GGC GTG-3’; *LD1F*: 5’-CAG ACA CCC TCA AAT TGT GCC-3’; *LD1R*: 5’-GGC ACT GCA CTG TTA ATA ATC T-3’; *LD2R*: 5’-CAG TGA CGC ACC CTG TGT TGT GA-3’(Sigma-Aldrich, St. Louis, MO USA). PCR reactions with all eleven primer pair combinations (Fig 1) were set in a final volume of 25 *μ*l: PCR reaction buffer [750 mM Tris–HCl (pH 8.8 at 25°C), 200 mM (NH_4_)_2_SO_4_, 0.1% Tween 20] (Thermo Scientific), 2.5 mM MgCl_2,_ 1.0 mM dNTPs, 0.5 μM each primer, 1 U of *DreamTaq* DNA Polymerase (Thermo Scientific) and 50 ng of gDNA. The temperature profile was: initial denaturation at 94°C for 4 min, 28 cycles (94°C for 20 s; 60°C for 30 s; 72°C for 1.5 min) and a final extension at 72°C for 10 min, in a Thermal Cycler 2720 (Applied Biosystems, Boston, CA, USA). Amplification products were separated, visualized and photo-documented as above.

### Validation by qPCR

For confirmation of the previous results, we selected sixteen individuals from the total sample, four per group: 0B, 1B, 2B and 3B chromosomes. The relative quantitation was used to evaluate the amplification of two selected B-specific fragments of 251 bp *LD1F- LD2R* and 321 bp *DD5F- DD5R*. We applied the *Rps18* gene, as an internal control, recommended as the most stable, thereby allowing direct comparison between liver samples [49]. A confirmation experiment with serial dilutions of gDNAs was done to demonstrate that the efficiencies of the target and internal control reactions were approximately equal. The *Rps18* sequence was amplified by the forward (5’-AGT TCC AGC ACA TTT TGC GAG-3’) and reverse (5’-TCA TCC TCC GTG AGT TCT CCA-3’) primers and the results are presented as the fold change relative to the calibrator sample (0B individuals). The quantitative PCR assays were set in duplicates for both target and control genes, for each sample. They were performed in a 10 μl reaction mixture containing 1× SYBR® Green PCR Master Mix (Applied Biosystems, Waltham, MA USA), 1.0 μM of each primer and 100 ng of DNA template, on an ABI Prism 7000 Sequence Detection System (Applied Biosystems, Waltham, MA USA). The temperature profile began with a holding stage at 50°C for 2 min, 95°C for 3 min, followed by 40 cycles: 95°C for 15 s, 60°C for 30 sec (fluorescence detection step) and 72.0°C for 30 sec.

We used the 2^−ΔΔCt^ method [50] for relative quantitation calculated from the threshold cycle (Ct) values. The experimental threshold was calculated based on the mean baseline fluorescence signal from cycle 3 to 15 plus 10 standard deviations. The point at which the amplification plot crosses this threshold (Ct) represents the exact cycle number and is inversely proportional to the number of target copies present in the initial sample. The ΔCt is calculated by normalizing the average Ct of the target B-variable fragments (251 bp *LD1F- LD2R* and 321 bp *DD5F- DD5R*) with the average Ct of the internal control (Ct target–Ct internal control). The ΔΔCt is then calculated by subtracting the ΔCt for the 0B sample from the corresponding ΔCt for the +B sample. The relative levels of the target amplification are expressed as the fold change relative to the calibrator sample. A relative quantity of 1 indicates no change in copy numbers. The following equation was used:

$$\mathrm{Relative Quantity}=2^{-\Delta \Delta Ct}$$

### Fluorescence In Situ Hybridization (FISH)

Metaphase preparations for FISH were made from primary spleen cell cultures from one 1B and two 0B animals. The cell lines were cultured for 48h at 37°C in RPMI Medium 1640 (Sigma-Aldrich, USA) enriched with 10% fetal bovine serum (Invitrogen, UK), Penicillin/Streptomycin 5,000 U/ml/5,000 g/ml (Invitrogen, UK) and stimulated by Lipopolysaccharides (Sigma-Aldrich, USA) and Concavalin A (Sigma-Aldrich, USA). Before harvest, the cells were treated with 10 μg/ml colchicine (final concentration) for 30 min. Preparation of metaphase chromosomes was made according to standard procedures that included a 20-minute hypotonic treatment in 0.56% KCl, fixation in 3:1 methanol/glacial acetic acid, followed by slide preparation and air drying. The number of B chromosomes was analyzed in at least 50 cells per sample and no variation was found in the number of B chromosomes between cells.

*DD5F- LD2R* 1459 bp product was amplified following the PCR conditions described above in a final volume of 50 *μ*l and labeled by Biotin Nick Translation Mix (Invitrogen, UK) following the manufacturer’s instructions to generate FISH probes. FISH was performed using a standard protocol [51] without pepsin pretreatment, with prolonged chromosome denaturation of 12 min at 74°C. After incubation overnight at 37°C, probes were detected in two ways: regular with Streptavidin-FITC (SA-FITC) and with Biotynilated Avidin/SA-FITC system.

Images were captured using Axioplan II Microscope (Carl Zeiss Jena GmbH, Germany) using with filter sets for DAPI and FITC. Image analysis was done using the ISIS digital FISH imaging system (Meta Systems Hard & Software GmbH, Althussheim, Germany). At least 60 metaphases per animal were analyzed for each applied probe.

## Results

### Karyotypes

Among the 387 mouse karyotypes examined, 262 were without Bs (0B) and 125 with Bs (+B). The karyotypes of 58 mice revealed one B chromosome, 16 had 2 Bs and six were with 3Bs. Six had one micro B (μB) chromosome and 39 were mosaics (Table 1). Two types of mosaics were recorded. Thus, nine mice had mosaics for one to three Bs (1-nBs), while thirty individuals had mosaics consisting of none to four Bs (0-nB).

### ISSR-PCR profiles

**Fig 2:**
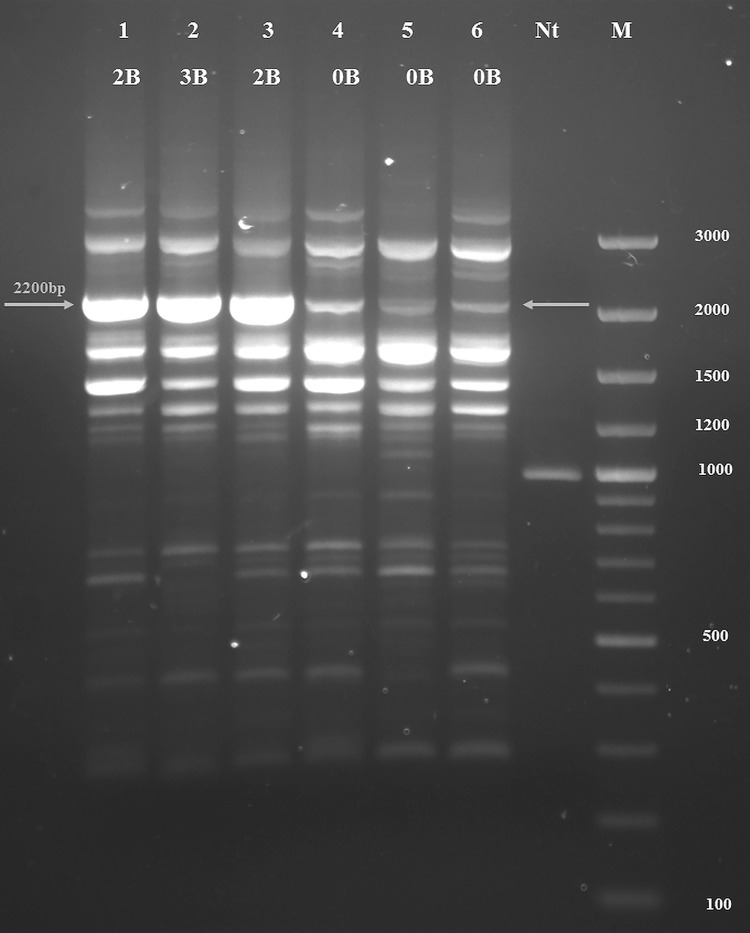
Electrophoretic scan of ISSR profiles amplified with primer (CAG)_4_AC. Lines 1, 2 and 3 obtained by +B gDNA; lines 4, 5 and 6 by 0B. Nt- negative control, M- GeneRuler 100 bp DNA Ladder Plus, Thermo Scientific.

Out of twelve optimized, six anchored primers gave specific and reproducible ISSR profiles of moderate complexity. Primer (CAG)_4_AC, with DNA fragments ranging from 290–3316 bp, produced a pattern specific for samples with Bs. Namely, samples with +B karyotypes gave a prominent 2200 bp electrophoretic band (B-marker) with strong quantitative discrepancy compared to samples with 0B karyotypes, which produced a thin DNA band of the same length (Fig 2). This result suggested significantly increased amplification of the 2200 bp DNA band in individuals with +B karyotypes. To verify the B-specific quantitative difference, we included more samples of *A*. *flavicollis*, captured at twenty different localities. Reproducibility of the ISSR profiles was confirmed with gDNA extracted from ear, tail and buccal swabs, which makes this method a noninvasive technique for B chromosome detection. The ISSR profiles and karyotypes of 387 individuals were compared for the presence/absence of B chromosomes (Table 1). Among 342 animals with 0, 1, 2 and 3B karyotypes total congruence with their ISSR profiles occurred for 330 (96.5%) (Table 1). For 262 samples with 0B karyotypes, 255 ISSR profiles confirmed the absence of Bs, but seven samples from three localities showed the presence of a B-marker. Among 58 samples with 1B, the presence of Bs was confirmed by ISSR in 53 cases, but five individuals from four localities gave a 0B ISSR result. Animals with two and three B karyotypes were identically recognized by both methods. The group of 45 animals with mosaic and μB karyotypes was considered separately (Table 1). Eight out of nine animals mosaic for different numbers of Bs (1-nBs) were confirmed by ISSR as +B karyotype (88.9%). In the group of animals which were mosaics consisting of zero to different numbers of Bs (0-nB) the ISSR method recognized fourteen as +B out of thirty (46.7%). Similarly, 50.0% of μB karyotypes were identified as +B. The overall method disagreement of this separate group was 44.45%.

### Homology between +B and 0B RFLP profiles

**Fig 3:**
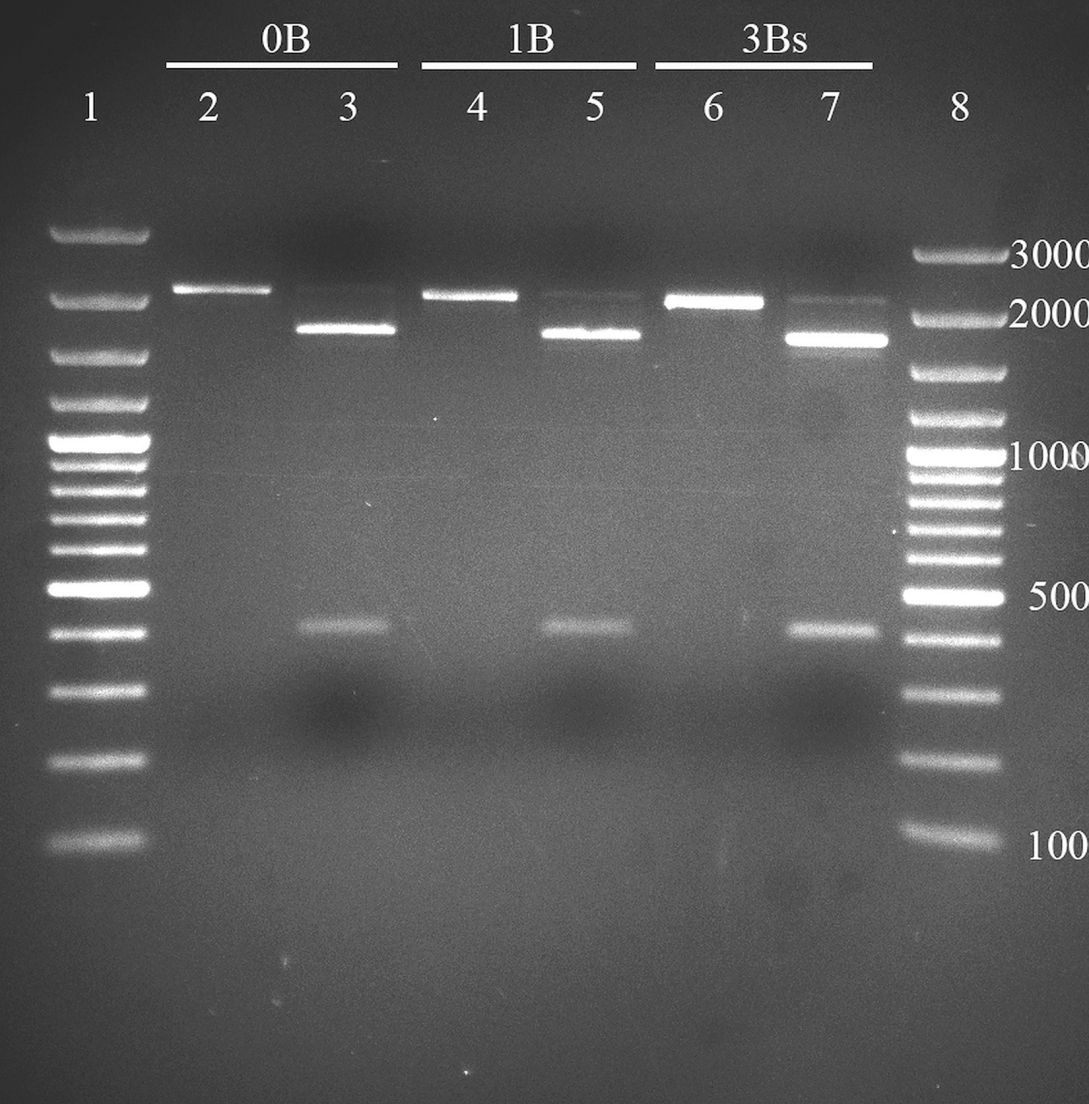
RFLP results of mixed digestion of 2200 bp DNA band with *Sac*I and *Hae*III. Lines 2, 4, 6—extracted and purified 2200 bp DNA bands without digestion; lines 3, 5, 7—digested 2200 bp bands; lines 1, 8- GeneRuler 100 bp DNA Ladder Plus, Thermo Scientific.

In order to explore preliminarily if 2200 bp bands consist of DNA molecules with the same sequence, we removed them from the agarose gel, purified and used them for RFLP analyses. For this approach we randomly chose five +B and five 0B animals from different localities. *Ecl*136II, *Sac*I and *Hae*III cleaved 2200 bp bands producing two fragments of 1800 bp and 400 bp; *Msp*I gave two bands of similar length ~950 bp; *Mbo*I provided a profile with three prominent bands: one ~550 bp, and two ~650 bp; *Apa*I, *Hind*III, *Bsp*120I, *Mfe*I, *Hpa*I, had no digestion activity; *Alu*I and *Mse*I produced highly complex, but unreadable profiles. There were no qualitative differences visible on agarose gel between +B and 0B digestion profiles, nor between samples with 1, 2 and 3 Bs (Fig 3). The obtained RFLP profiles indicated that the amplified 2200 bp bands consisted of amplicons with highly similar nucleotide sequences.

### B-marker matches to serine/threonine-protein kinase VRK1

The B-specific 2200 bp ISSR amplicon obtained from gDNA of a random animal with 1B karyotype was cloned and sequenced in both directions and is presented in Fig 1. The sequencing result was confident and completely identical among all ten clones in both, 991 nt at the 5’-end and 995 nt at the 3’-end, but around 200 bp internal segment was not clearly read by the sequencing. A comprehensive search in the NCBI GenBank database was made with BLASTN 2.2.31 [52,53]. The 995 bp 3’-end DNA sequence showed similarity with *serine/threonine-protein kinase VRK1 isoform a* and *b* genomic sequences, *Mus musculus* strain mixed chromosome 12, alternate assembly Mm_Celera Sequence ID:ref |AC_000034.1| with 98% identities. The 991 bp 5’-end DNA sequence showed 82% identities with *serine/threonine-protein kinase VRK1* isoform *a* and *b* genomic sequences, *Mus musculus* strain mixed chromosome 12, alternate assembly Mm_Celera Sequence ID: ref |AC_000034.1| (S1 Fig).

### Primers designed from B-marker sequence

**Fig 4:**
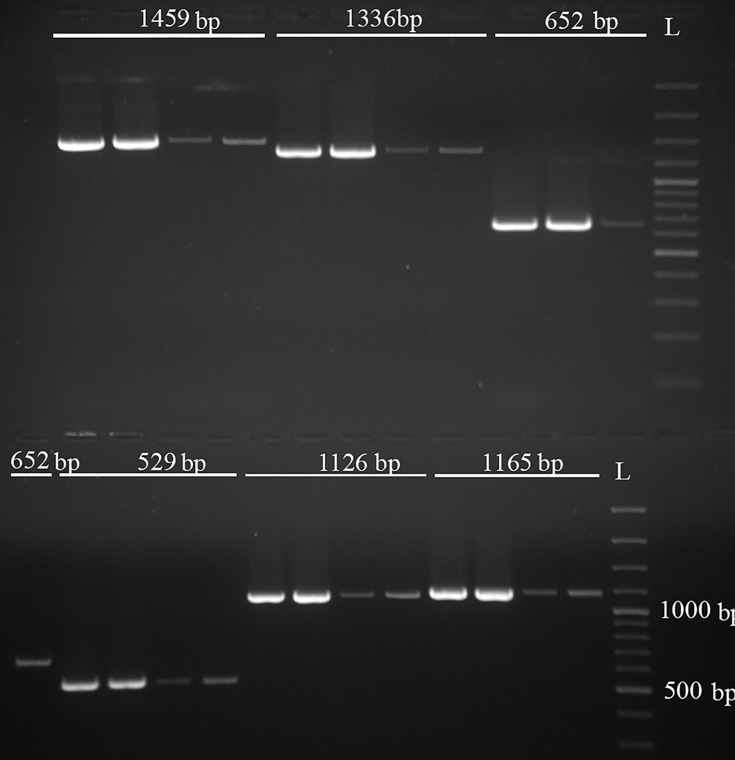
Electrophoretic scan of six PCR products of different lengths. +B/0B quantitative discrepancy, the first two templates +B and the second two templates 0Bs. L- GeneRuler 100 bp DNA Ladder Plus (Thermo Scientific).

**Fig 5:**
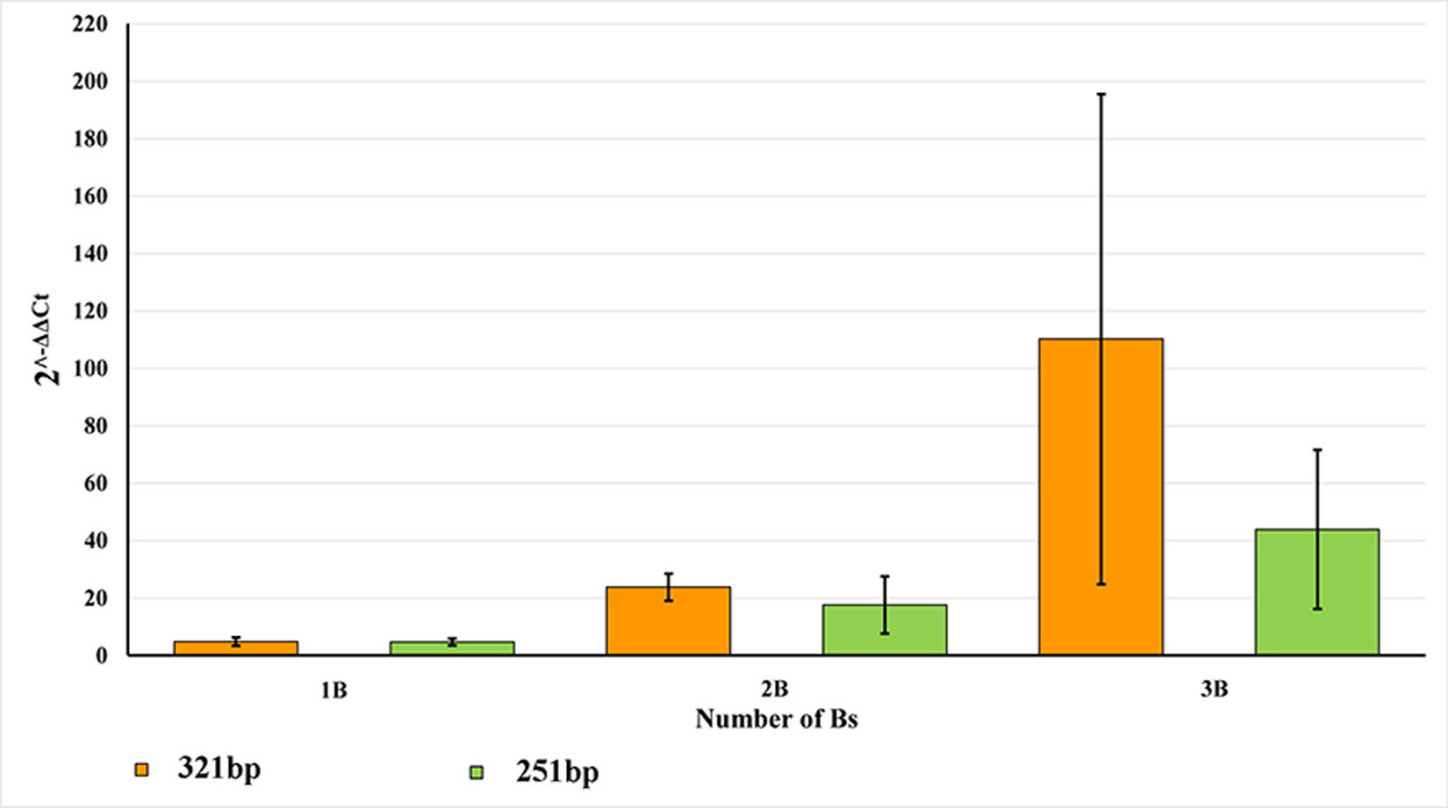
Results of the Calculation of Imported Threshold Cycle (Ct) Data for Two Target Sequences. Orange 321 bp amplified with *DD5F - DD5R* primer pair; green 251 bp amplified with *LD1F - LD2R*.

Primers for an End Point PCR were designed from both the 991 nt region at the 5’-end and the 995 nt region at the 3’-end of the 2200 bp B-specific sequence (Fig 1). They produced eleven specific PCR products amplified from gDNA, which were assigned as: *LD1F- LD1R* 128 bp, *LD1F- LD2R* 251 bp, *SR1F- SR1R* 319 bp, *DD5F- DD5R* 321 bp, *SR1F- SR2R* 359 bp, *SR1F- LD1R* 529 bp, *SR1F-LD2R* 652 bp, *DD5F- SR1R* 1126 bp, *DD5F- SR2R* 1165 bp, *DD5F- LD1R* 1336 bp and *DD5F*- *LD2R* 1459 bp (Fig 4). The quantitative difference in amplicons produced from +B/0B gDNA as template was visualized in agarose gel and confirmed in all End Point PCR runs (Figs 4 and 5).

### qPCR confirms +B/0B discrepancy

Two primer pairs *LD1F- LD2R* (251 bp) and *DD5F - DD5R* (321 bp) with the most suitable amplicons (their length and location on different (5’ and 3’ end) of a 2200 bp amplicon) were selected for qPCR (Fig 5). The results of the calculation of imported threshold cycle (Ct) data for target sequences 321 bp and 251 bp are shown in S1 Table and Fig 5. It is evident that the 321 bp amplicon was 4.82 fold increased in 1B animals, 23.84 fold in 2Bs and even 110.24 fold in 3B individuals. The 251 bp amplicon was highly similarly increased in 1B (4.72 fold) and 2Bs animals (17.61 fold) but less so in 3Bs samples (43.94 fold).

## Fluorescent signal on Bs

**Fig 6:**
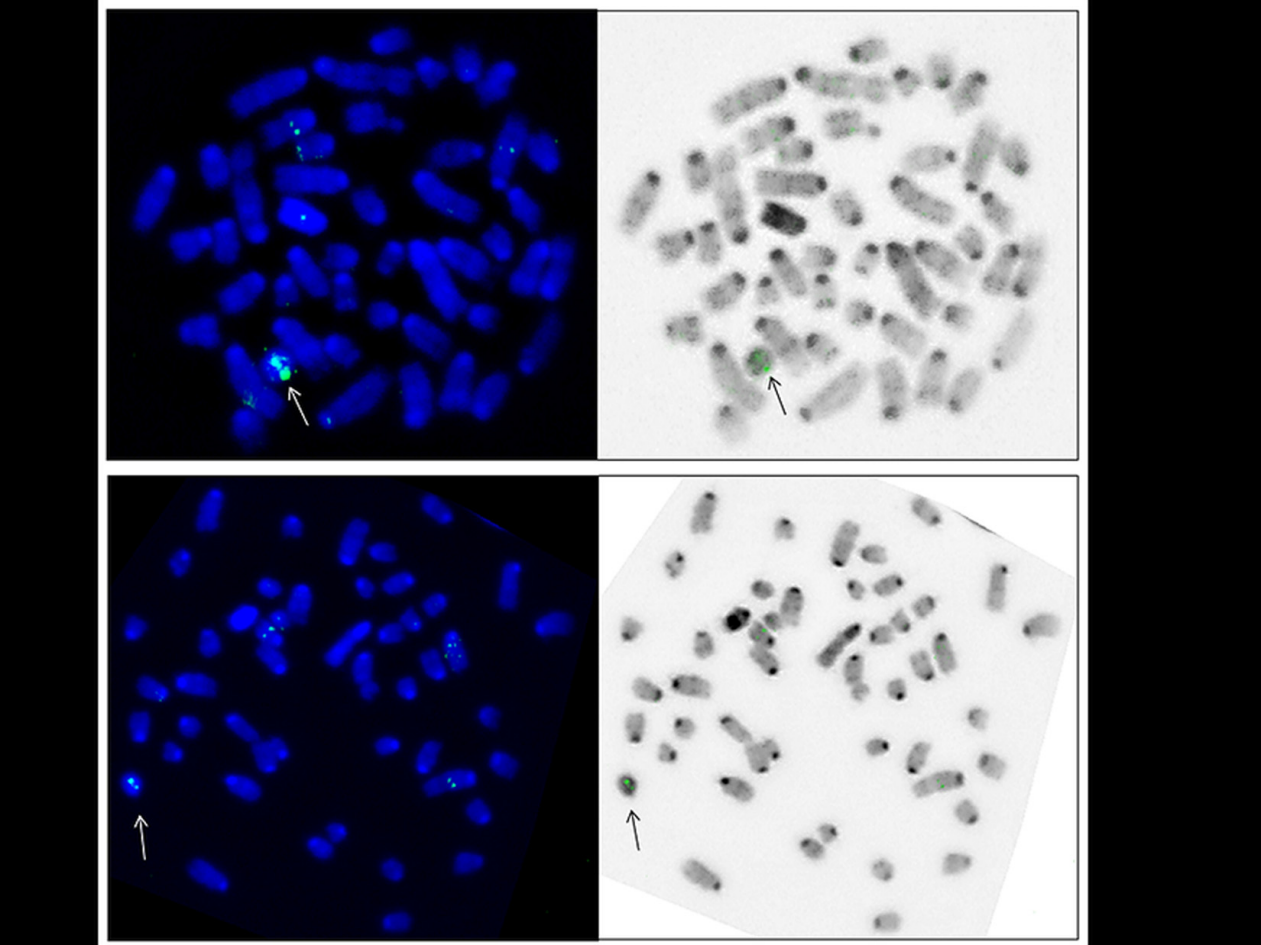
Hybridization of B-marker probe in two metaphases in 1B *A*. *flavicollis*. B chromosome is marked with an arrow.

The signals on the B-chromosomes were seen on 15% of analyzed metaphases, always located in the same chromosomal region (Fig 6). All samples (one 1B and two 0B), had background signals on random chromosomes with different locations between metaphases. There was no significant difference between regular and detection with biotinylated antiavidin.

## Discussion

In quest of a distinctive B marker among repetitive sequences, frequently reported in B chromosomes of various species [13,26–32], clear quantitative difference was observed in a 2200 bp electrophoretic band (Fig 2) between +B and 0B samples. Comparison of karyotypes from 342 animals (excluding mosaic and μB animals) with their respective ISSR profiles showed total congruence of 96.5% (Table 1). This highly recommends the ISSR method developed here for detection of Bs in live individuals with unknown karyotypes. On the contrary, the group of mosaic and μB animals was complex and comprised different karyotypes and therefore less congruence (55.5%) was expressed between the molecular and cytogenetic approaches (Table 1).

There are two possible explanations, which do not exclude each other, for this method disagreement. The first is the question of tissue and cell mosaicism. Namely, Bs may be absent from some tissues, including the liver, and consequently may not be recognized by the ISSR method in all samples. This could be why a B-marker may occur in the ISSR profile of mice with an 0B karyotype or be absent in 1B and mosaic animals. Tissue mosaicism has already been described in this species [54]. Also, the proportions of 0B/+B cells in both bone marrow and liver could be inclined in the direction of 0B, which may explain the greatest disagreement in 0-nB cases. The second explanation is the presence of different types of B chromosomes, as proposed by [55]. The authors explored the meiotic behavior of Bs in *A*. *flavicollis*, and found that when two Bs are present they could appear as univalents or form bivalents, indicating the occurrence of at least two types of Bs. The absence of a B-specific marker in 1B samples may be the consequence of both, tissue mosaicism and the existence of different B chromosomes with variable nucleotide sequences in *A*. *flavicollis*. The fact that in our analysis the B-marker was recorded in every sample (Table 1) with 2B and 3B karyotypes means that at least one of these B chromosomes carried a B-specific sequence, which may indicate that Bs with different molecular structures could reside in the same individual.

Further validation of the increased amplification of B-markers in ISSR profiles of 1, 2 and 3 Bs relative to 0B gDNA by qPCR showed a clear +B/0B quantitative shift. Relative amplification of the 251 bp and 321 bp PCR products (Fig 5) was similarly increased in 1B samples (4.80 and 4.72 fold, respectively). This finding, together with the ISSR profiles and End Point PCR results (Figs 2 and 4), indicates that sequences detected on Bs may be amplified on the B chromosome, as also reported by [56,57]. The evident discrepancy in amplification level of the two targeted sequences from B-markers in animals with 2 and 3 Bs (S1 Table and Fig 5), high standard deviation for the average 2^-ΔΔCt^ in 3Bs group, along with the meiotic behavior of Bs in *A*. *flavicollis* mentioned above, confirms our assumption that different types of Bs with variable molecular composition may exist in the same individual and between individuals of this species. Bs of many species contain sequences that originated from one or more As [32,58]. In a single individual Bs could have different origins and evolutionary pathways, i.e. amplification and/or deletion of some parts of gene sequences. Studies on Bs from different mammalian species [6,8,59] showed that Bs frequently carry duplicated gene segments, some with variable blocks sizes in different Bs and show significant similarity with homologous autosomal regions. Correspondingly, an accumulation of B-enriched sequences, present in As in a low copy number, was found in rye [60] indicating an intraspecific origin of Bs. However, the same B enriched sequences were observed in other species from the same genus, as well [60]. Similarly, in this study we included in our ISSR analysis two specimens of *A*. *sylvaticus*, one with +B and one with an 0B karyotype, and gained the same result as for *A*. *flavicollis*. Namely, an amplified B-marker was present in the +B and a weak band in the 0B individual. Although found in very low frequencies in *A*. *sylvaticus*, compared to the common occurrence in *A*. *flavicollis*, Bs in these two species could arise in a similar fashion, or originate from the same region of the standard chromosomal complement.

Considering our result from qPCR analysis and studies on Bs from different mammalian species [6,8,59], in 1B sample target sequence could be 10 times amplified, probably in the same block. Even multiplied, FISH signal is approximately 15000 bp long, which still represents very small size of the probe covered region [61]. That could be the reason why the signal was visible on 15% of all analyzed metaphases of 1B animal. Nevertheless, this result is sufficient to prove that B-marker is actually located on B chromosomes. According to GeneBank data, *serine/threonine-protein kinase VRK1* gene sequence is located in one copy on chromosome No. 12 in *Mus musculus*, which corresponds to chromosome No. 6 in *A*. *flavicollis* [62]. Although presence of 2200 bp sequence is estimated on A chromosomes, a lack of signal is expected, because a 1459 bp probe covered region is under the detection limit of these methods.

Even though FISH could not demonstrate the origin of B-marker from A chromosomes, qualitative analysis of 2200 bp bands between +B and 0B samples by RFLP showed high sequence similarity in +B and 0B digestion profiles (Fig 3) and indicated that the B- marker could be consisted of the same amplicons. Furthermore, this result substantiates the numerous times reported theory that Bs originate from the standard chromosomal complement. Derivation of Bs in *A*. *flavicollis* through polysomy of small autosomes was proposed long ago by [20]. The authors further hypothesized that their emergence is followed by heterochromatinization, similarly to the mammalian X chromosome, which is invisible with C-banding. A certain form of homology at the molecular level was confirmed by [63].

Traditionally, B chromosomes were considered to be inert genomic elements. The first discovery of protein-coding genes in Bs of the fungus *Nectria haematococca* [64] was followed by a growing number of reports describing copies of protein coding genes and pseudogenes on Bs [56,58,59,65–67]. Latter were introduced new techniques that considerably altered our view of the molecular structure of Bs using localization of BAC (bacterial artificial chromosome) clones and sequencing of isolated B-specific DNA fragments [28]. This led to the identification of non-repetitive sequences on Bs. Further research confirmed the transcription of rRNA genes [68,69] and repetitive DNA with similarity to mobile elements [70] residing in B chromosomes. Recently, [6] showed that protein-coding genes on B chromosomes are transcribed from Bs of the Siberian roe deer, *Capreolus pygargus*. It was demonstrated [71] that 15% of the pseudogene-like fragments on Bs in a plant model are transcribed in a tissue-type and genotype specific manner. All these largely modify the conventional interpretation of totally silent B chromosomes and necessitate new research in other species.

The obtained parts of sequences of the B- marker showed a high level of identity with the gene sequences of *serine/threonine-protein kinase VRK1*, which plays an essential and evolutionarily conserved role in gametogenesis and fertility in worms, flies and mammals [72]. It is highly expressed in the testis, also expressed in the liver, kidney and muscle, but weakly in the thymus, bone marrow and spleen. Its exclusive localization in nuclei may suggest a potential role in DNA/RNA metabolism and the cell cycle [73]. Even though we detected part of the *VRK1* gene sequence by ISSR-PCR screening, further research is necessary to show the presence of the complete gene on Bs and its functional activity. Our results are comparable with the findings in the Siberian roe deer, *Capreolus pygargus* [6], who discovered transcriptional activity of *TNNI3K* Interacting Kinase gene, which codes for a protein with serine/threonine activity located on Bs. These authors found on Bs a 2 Mbp region covering three genes (*FPGT*, *LRRIQ3* and *TNNI3K*).Similarly, the proto-oncogene c-KIT was reported in Bs of three different canids, *Vulpes vulpes*, *Nyctereutes procyonoides* and *N*. *p*. *viverrinus* as well as on Bs of the cervid *Mazama gouazoubira* [8,59,66,], but the preliminary study by Real Time PCR of exon 1 excluded the presence of c-KIT proto-oncogene on Bs in *A*. *flavicollis* [74].

Numerus genes crucial in proliferation and cell differentiation, involved in oncogenesis, sex determination and cell division [56,57,75] found on Bs from phylogenetically greatly distant vertebrate species, argue that there is a similar mechanism for emergence and conservation of supernumeraries, possibly from inherently unstable genomic parts that may represent recombination or evolutionary hotspots [8]. Considering the reported similarities among Bs from phylogenetically highly distant species stated above, partial homology in nucleotide sequences of Bs is not surprising, but should be further explored.

In conclusion, screening of SSR sequences with primer (CAG)_4_AC proved to be simple, reliable, and most of all a highly specific method for detection of B presence in *A*. *flavicollis*, providing an opportunity for noninvasive sampling from natural populations and inheritance studies of Bs. The high level of identity of the B-marker with *serine/threonine-protein kinase* (*VRK1*) gene sequences adds to the growing list of protein-coding genes recorded in B chromosomes. Further research is necessary to infer the origin, heritability, transcriptional activity and potential effects of Bs in *A*. *flavicollis* and thus make a contribution to elucidation of vertebrate genome evolution.

## Supporting Information

S1 Fig Results of BLAST homology search in the NCBI GenBank database for B-variable DNA sequence. Alignments with the highest degree of similarity are shown: a) Query 998 bp 3’-end DNA fragment, Sbjct sequences from databases. 998 bp 3’-end DNA sequence showed similarity with serine/threonine-protein kinase VRK1 isoform a and serine/threonine-protein kinase VRK1 isoform b genomic sequences, Mus musculus strain mixed chromosome 12, alternate assembly Mm_Celera Sequence ID: ref|AC_000034.1| b)Mus musculus vaccinia related kinase 1 (Vrk1), transcript variant X7, mRNA, with 98% identities; c) 991 bp 5’-end DNA sequence showed 82% similarity with serine/threonine-protein kinase VRK1 isoform a and serine/threonine-protein kinase VRK1 isoform b genomic sequences, Mus musculus strain mixed chromosome 12, alternate assembly Mm_Celera Sequence ID: ref|AC_000034.1|.
(DOC)

S1 Table The results of qPCR analysis. (DOCX)

## References

[1] Beukeboom LW. Bewildering Bs: an impression of the 1st B-chromosome conference. Heredity. 1994; 73:328–336.

[2] Randolph LF. Types of supernumerary chromosomes in maize. Anat Rec. 1928; 41:102.

[3] Jones RN, Rees H. B Chromosomes 1st ed. Academic Press; 1982.

[4] Jones N, Houben A. B chromosomes in plants: escapees from the A chromosome genome? Trends Plant Sci. 2003; 8:417–423. 13678908 10.1016/S1360-1385(03)00187-0

[5] Houben A, Banaei‑Moghaddam AM, Klemme S, Timmis JN. Evolution and biology of supernumerary B chromosomes. Cell Mol Life Sci. 2013; 71:467–478. 10.1007/s00018-013-1437-7 23912901 PMC11113615

[6] Trifonov VA, Dementyeva PV, Larkin DM, O’Brien PCM, Perelman PL, Yang F, et al Transcription of a protein-coding gene on B chromosomes of the Siberian roe deer (Capreolus pygargus). BMC Biol. 2013; 11:90 10.1186/1741-7007-11-90 23915065 PMC3751663

[7] Vujošević M, Blagojević J. B chromosomes in populations of mammals. Cytogenet Genome Res. 2004; 106:247–256. 15292599 10.1159/000079295

[8] Makunin AI, Dementyeva PV, Graphodatsky AS, Volobouev VT, Kukekova AV, Trifonov VA. Genes on B chromosomes of vertebrates. Mol Cytogenet. 2014; 7:99 10.1186/s13039-014-0099-y 25538793 PMC4274688

[9] Wilson DE, Reeder DM. Mammal Species of the World: A Taxonomic and Geographic Reference 3rd ed. Baltimore: Johns Hopkins University Press; 2005.

[10] Kartavtseva IV. Karyosystematics of wood and field mice (Rodentia, Muridae). Vladivostok: Dalnauka. 2002; pp. 140.

[11] Matsubara K, Yamada K, Umemoto S, Tsuchiya K, Ikeda N, Nishida C, et al Molecular cloning and characterization of the repetitive DNA sequences that comprise the constitutive heterochromatin of the A and B chromosomes of the Korean field mouse (Apodemus peninsulae, Muridae, Rodentia). Chromosome Res. 2008; 16:1013–1026. 10.1007/s10577-008-1259-x 18949567

[12] Obara Y, Sasaki S. Fluorescent approaches on the origin of B chromosomes of *Apodemus argenteus hokkaidi*. Chrom Sci. 1997; 1:1–5.

[13] Karamysheva TV, Andreenkova OV, Bochkaerev MN, Borissov YM, Bogdanichkova N, Borodin PM. B chromosomes of Korean field mouse Apodemus peninsulae (Rodentia, Murinae) analysed by microdissection and FISH. Cytogenet Genome Res. 2002; 96(1–4):154–60. 12438792 10.1159/000063027

[14] Serizawa K, Suzuki H, Tsuchiya K. A phylogenetic view on species radiation in *Apodemus* inferred from variation of nuclear and mitochondrial genes. Biochem Genet. 2000; 38:27–40. 10862357 10.1023/a:1001828203201

[15] Kartavtseva IV. Description of B Chromosomes in the Karyotype of Field Mouse Apodemus agrarius. Tsitol Genet. 1994; 28(2):96–97. 7941023

[16] Zima J, Macholan M. B chromosome in the wood mice (genus Apodemus). Acta Theriol Suppl. 1995; 3:75–86.

[17] Kartavtseva IV, Roslik GV. A complex B chromosome system in the Korean field mouse, Apodemus peninsulae. Cytogenet Genome Res. 2004; 106:271–278. 15292602 10.1159/000079298

[18] Kral B, Zima J, Herzig-Strachil B, Štreba O. Karyotypes of certain small mammals from Austria. Folia Zool. 1979; 28:5–11.

[19] Zima J. Chromosomes of certain small mammals from southern Bohemia and the Sumava mts. Folia Zool. 1984; 33:133–141.

[20] Vujošević M, Živković S. Numerical cromosome polymorphism in Apodmeus flavicollis and *A*. *sylvaticus* (Mammalia: Rodentia) caused by supernumerary chromosomes. Acta Vet. 1987; 37(2–3):81–92.

[21] Vujošević M, Blagojević J, Radosavljević J, Bejaković D. B chromosome polymorphism in populations of *Apodemus flavicollis* in Yugoslavia. Genetica. 1991; 83:167–170.

[22] Vujošević M, Blagojević J. Does environment affect polymorphism of B chromosomes in the yellow-necked mouse *Apodemus flavicollis*? Z Säugetierkunde. 2000; 65: 313–317.

[23] Blagojević J, Vujošević M. The role of B chromosomes in the population dynamics of yellow necked wood mice *Apodemus flavicollis* (Rodentia, Mammalia). Genome. 1995; 38:472–478. 7557359 10.1139/g95-062

[24] Jojić V, Blagojević J, Vujošević M. B chromosomes and cranial variability in yellow-necked field mice (*Apodemus flavicollis*). J Mammol. 2011; 92(2):396–406.

[25] Adnađević T, Bugarski-Stanojević V, Blagojević J, Stamenković G, Vujošević M. Genetic differentiation in populations of the yellow-necked mouse, *Apodemus flavicollis*, harboring B chromosomes in different sequences. Popul Ecol. 2012; 54:537–548.

[26] Houben A, Leach CR, Verlin D, Rofe R, Timmis JN. A repetitive DNA sequence common to the different B chromosomes of the genus *Brachycome*. Chromosoma. 1997; 106(8):513–519. 9426283 10.1007/pl00007689

[27] Yang F, O’Brien PCM, Wienberg J, Neitzel H, Lin CC, Ferguson-Smith MA. Chromosomal evolution of the Chinese muntjac (Muntiacus reevesi). Chromosoma. 1997; 106:37–43. 9169585 10.1007/s004120050222

[28] Trifonov VA, Perelman PL, Kawada S-I, Iwasa MA, Oda S-I, Graphodatsky AS. Complex structure of B-chromosomes in two mammalian species: Apodemus peninsulae (Rodentia) and Nyctereutes procyonoides (Carnivora). Chromosome Res. 2002; 10:109–116. 11993931 10.1023/a:1014940800901

[29] Jesus CM, Galetti PM Jr, Valentini SR, Moreira-Filho O. Molecular characterization and chromosomal localization of two families of satellite DNA in *Prochilodus lineatus* (Pisces, Prochilodontidae), a species with B chromosomes. Genetica. 2003; 118:25–32. 12737143 10.1023/a:1022986816648

[30] Rubtsov NB, Karamysheva TV, Andreenkova OV, Bochkaerev MN, Kartavtseva IV, Roslik GV, et al Comparative analysis of micro and macro B chromosomes in the Korean field mouse Apodemus peninsulae (Rodentia, Murinae) performed by chromosome microdissection and FISH. Cytogenet Genome Res. 2004; 106:289–294. 15292605 10.1159/000079301

[31] Bugrov AG, Karamysheva TV, Pyatkova MS, Rubtsov DN, Andreenkova OV, Warchałowska-Śliwa E. et al B Chromosomes of the *Podisma sapporensis* Shir. (Orthoptera, Acrididae) Analysed by Chromosome Microdissection and FISH. Folia Biol-Krakow. 2003; 51:1–2. 14686642

[32] Bugrov AG, Karamysheva TV, Perepelov EA, Elisaphenko EA, Rubtsov DN, Warchałowska-Śliwa E, et al DNA content of the B chromosomes in grasshopper Podisma kanoi Storozh. (Orthoptera, Acrididae). Chromosome Res. 2007; 15:315–325. 17406993 10.1007/s10577-007-1128-z

[33] Silva DMZdA, Pansonato-Alves JC, Utsunomia R, Araya-Jaime C, Ruiz-Ruano FJ, et al Delimiting the Origin of a B Chromosome by FISH Mapping, Chromosome Painting and DNA Sequence Analysis in Astyanax paranae (Teleostei, Characiformes). PLoS ONE. 2014; 9(4): e94896 10.1371/journal.pone.0094896 24736529 PMC3988084

[34] Yamada K, Nishida-Umehara C, Matsuda Y. A new family of satellite DNA sequences as a major component of centromeric heterochromatin in owls (Strigiformes). Chromosoma. 2004; 112:277–287. 14997323 10.1007/s00412-003-0267-z

[35] Rubtsov NB, Kartavtseva IV, Roslik GV, Karamysheva TV, Pavlenko MV, Iwasa MA, et al Features of the B Chromosome in Korean Wood Mice Apodemus peninsulae (Thomas, 1906) from Transbaikalia and the Far East Identified by the FISH Method. Russ J Genet. 2015 51(3):278–288. 26027373

[36] Katti MV, Ranjekar PK, Gupta VS. Differential Distribution of Simple Sequence Repeats in Eukaryotic Genome Sequences. Mol Biol Evol. 2001; 18(7):1161–1167. 11420357 10.1093/oxfordjournals.molbev.a003903

[37] Zietkiewicz E, Rafalski A, Labuda D. Genome fingerprinting by simple sequence repeat (SSR)-anchored polymerase chain reaction amplification. Genomics. 1994; 20:176–183. 8020964 10.1006/geno.1994.1151

[38] Bornet B, Branchard M. Nonanchored Inter Simple Sequence Repeat (ISSR) Markers: Reproducible and Specific Tools for Genome Fingerprinting. Plant Mol Biol Rep. 2001; 19:209–215.

[39] Wang HZ, Wu ZX, Lu JJ, Shi NN, Zhao Y, Zhang ZT, et al Molecular diversity and relationships among *Cymbidium goeringii* cultivars based on inter simple sequence repeat (ISSR) markers. Genetica. 2009; 136:391–399. 10.1007/s10709-008-9340-0 19085060

[40] Bugarski-Stanojević V, Blagojević J, Stamenković G, Adnađević T, Giagia-Athanasopoulou EB, Vujošević M. Comparative study of the phylogenetic structure in six *Apodemus* species (Mammalia, Rodentia) inferred from ISSR-PCR data. Syst Biodivers. 2011; 9:95–106.

[41] Bugarski-Stanojević V, Blagojević J, Adnađević T, Jovanović V, Vujošević M. Identification of the sibling species *Apodemus sylvaticus* and *A*. *flavicollis* (Rodentia, Muridae)–comparison of molecular methods. Zool Anz. 2013; 252:579–587.

[42] McClelland M, Welsh J. DNA fingerprinting by arbitrarily primed PCR. PCR Meth Appl. 1994; 4:59–65. 10.1101/gr.4.1.s59 9018327

[43] Tyler KD, Wang G, Tyler SD, Johnson WM. Factors affecting reliability and reproducibility of amplification-based DNA fingerprinting of representative bacterial pathogens. J Clin Microbiol. 1997; 35:339–346. 9003592 10.1128/jcm.35.2.339-346.1997 PMC229576

[44] Cobb BD, Clarkson JM. A simple procedure for optimizing the polymerase chain reaction (PCR) using modified Taguchi methods. Nucleic Acids Res. 1994; 22: 3801–3805. 7937094 10.1093/nar/22.18.3801 PMC308365

[45] Hall TA. BioEdit: a user-friendly biological sequence alignment editor and analysis program for Windows 95/98/NT. Nucl Acids Symp Ser. 1999; 41:95–98.

[46] Tamura K, Stecher G, Peterson D, Filipski A, Kumar S. MEGA6: Molecular Evolutionary Genetics Analysis version 6.0. Mol Biol Evol. 2013; 30:2725–2729. 10.1093/molbev/mst197 24132122 PMC3840312

[47] Koressaar T, Remm M. Enhancements and modifications of primer design program Primer3. Bioinformatics. 2007; 23(10):1289–91. 17379693 10.1093/bioinformatics/btm091

[48] Owczarzy R, Tataurov AV, et al IDT SciTools: a suite for analysis and design of nucleic acid oligomers. Nucleic Acids Res. 2008; 36(2):163–169. 10.1093/nar/gkn198 PMC2447751 18440976

[49] Axtner J, Sommer S. Validation of internal reference genes for quantitative real-time PCR in a non-model organism, the yellow-necked mouse, Apodemus flavicollis. BMC Res Note 2009; 2:264. 10.1186/1756-0500-2-264 PMC2804578 20030847

[50] Livak KJ, Schmittgen TD. Analysis of relative gene expression data using Real-Time quantitative PCR and the 2− Δ ΔCt method. Methods. 2001; 25:402–408. 11846609 10.1006/meth.2001.1262

[51] Liehr T, Pellestor F. Molecular Cytogenetics: The Standard FISH and PRINS Procedure In: Liehr T, editor. Fluorescence In Situ Hybridization (FISH)—Application Guide. Springer-Verlag Berlin Heidelberg; 2009 pp. 23–34.

[52] Zhang Z, Schwartz S, Wagner L, Miller W. A greedy algorithm for aligning DNA sequences. J Comput Biol. 2000; 7(1–2):203–14. 10890397 10.1089/10665270050081478

[53] Morgulis A, Coulouris G, Raytselis Y, Madden TL, Agarwala R, Schäffer AA. Database Indexing for Production MegaBLAST Searches. Bioinformatics. 2008; 24:1757–1764. 10.1093/bioinformatics/btn322 18567917 PMC2696921

[54] Vujošević M, Jojić V, Blagojević J. Intra Individual Variation in the Number of B Chromosomes in the Yellow-necked Mouse, Apodemus flavicollis (Mammlia, Rodentia). Folia Biol. 2005; 53: 1–2. 16212112

[55] Vujošević M, Radosavljević J, Živković S. Mitotic Behaviour of B Chromosomes in Yellow Necked Mouse Apodemus flavicollis. Arch Biol Sci. 1990; 42(1–2):39–42.

[56] Yoshida K, Terai Y, Mizoiri S, Aibara M, Nishihara H, Watanabe M, et al B chromosomes have a functional effect on female sex determination in Lake Victoria cichlid fishes. PLoS Genet. 2011; 7: e1002203 10.1371/journal.pgen.1002203 21876673 PMC3158035

[57] Valente GT, Conte MA, Fantinatti BEA, Cabral-de-Mello DC, Carvalho RF, Vikari MR, et al Origin and Evolution of B Chromosomes in the Cichlid Fish *Astatotilapia latifasciata* Based on Integrated Genomic Analyses. Mol Biol Evol. 2014; 31(8):2061–2072. 10.1093/molbev/msu148 24770715

[58] Martis MM, Klemme S, Banaei-Moghaddam AM, Blattner FR, Macas J, Schmutzer T, et al Selfish supernumerary chromosome reveals its origin as a mosaic of host genome and organellar sequences. PNAS. 2012; 109(33)13343–13346. 10.1073/pnas.1204237109 22847450 PMC3421217

[59] Graphodatsky AS, Kukekova AV, Yudkin DV, Trifonov VA, Vorobieva NV, Beklemisheva VR, et al The proto-oncogene c-KIT maps to canid B-chromosomes. Chromosome Res. 2005; 13:113–122. 15861301 10.1007/s10577-005-7474-9

[60] Klemme S, Banaei-Moghaddam AM, Macas J, Wicker T, Novak P, Houben A. High-copy sequences reveal distinct evolution of the rye B chromosome. New Phytol. 2013; 199:550–558. 10.1111/nph.12289 23614816

[61] Navin N, Grubor V, Hicks J, Leibu E, Thomas E, Troge J, et al PROBER: oligonucleotide FISH probe design software. Bioinformatics. 2006; 22(19): 2437–2438. 16740623 10.1093/bioinformatics/btl273

[62] Matsubara K, Nishida-Umehara C, Tsuchiya K, Nukaya D, Matsuda Y. Karyotypic evolution of Apodemus (Muridae Rodentia) inferred from comparative FISH analyses. Chromosome Res. 2004; 12:383–395. 15241017 10.1023/B:CHRO.0000034103.05528.83

[63] Tanić N, Dedović N, Vujošević M, Dimitrijević B. DNA profiling of B-chromosomes from the yellow-necked mouse *Apodemus flavicollis* (Rodentia, Mammalia). Genome Res. 2000; 10:55–61. 10645950 PMC310505

[64] Miao VP, Covert SF, Van Etten HD. A fungal gene for antibiotic resistance on a dispensable (“B”) chromosome. Science. 1991; 254:1773–1776. 1763326 10.1126/science.1763326

[65] Han Y, Liu X, Benny U, Kistler HC, Vanetten HD. Genes determining pathogenicity to pea are clustered on a supernumerary chromosome in the fungal plant pathogen Nectria haematococca. Plant J. 2001; 3:305–314. 10.1046/j.1365-313x.2001.00969.x 11208022

[66] Yudkin DV, Trifonov VA, Kukekova AV, Vorobieva NV, Rubtsova NV, Yang F, et al Mapping of KIT adjacent sequences on canid autosomes and B chromosomes. Cytogenet Genome Res. 2007; 116(1–2):100–103. 17268185 10.1159/000097424

[67] Lamatsch DK, Trifonov V, Schories S, Epplen JT, Schmid M, Schartl M. Isolation of a cancer-associated microchromosome in the sperm-dependent parthenogen Poecilia formosa. Cytogenet Genome Res. 2011; 135:135–142. 10.1159/000331271 21952475

[68] Leach CR, Houben A, Bruce F, Pistrick K, Demidov D, et al Molecular evidence for transcription of genes on a B chromosome in Crepis capillaris. Genetics. 2005; 171: 269–278. 15956665 10.1534/genetics.105.043273 PMC1456518

[69] Ruìz-Estévez M, López-León MD, Cabrero J, Camacho JPM. B Chromosome Ribosomal DNA Is Functional in the Grasshopper Eyprepocnemis plorans. PLoS ONE. 2012; 7(5):e36600 10.1371/journal.pone.0036600 22570730 PMC3343036

[70] Carchilan M, Kumke K, Mikolajewski S, Houben A. Rye B chromosomes are weakly transcribed and might alter the transcriptional activity of A chromosome sequences. Chromosoma. 2009; 118:607–616. 10.1007/s00412-009-0222-8 19575213

[71] Banaei-Moghaddam AM, Meier K, Karimi-Ashtiyani R, Houben A. Formation and Expression of Pseudogenes on the B Chromosome of Rye. The Plant Cell. 2013; 25:2536–2544. 10.1105/tpc.113.111856 23839789 PMC3753381

[72] Wiebe MS, Nichols RJ, Molitor TP, Lindgren JK, Traktman P. Mice Deficient in the Serine/Threonine Protein Kinase VRK1 Are Infertile Due to a Progressive Loss of Spermatogonia. Biol Reprod. 2010; 82:182–193. 10.1095/biolreprod.109.079095 19696012 PMC2802121

[73] Zelko I, Kobayashi R, Honkakoski P, Negishi M. Molecular cloning and characterization of a novel nuclear protein kinase in mice. Arch Biochem Biophys. 1998; 352(1):31–6. 9521809 10.1006/abbi.1998.0582

[74] Rajičić M, Adnađević T, Stamenković G, Blagojević J, Vujošević M. Screening of B chromosomes for presence of two genes in yellow-necked mice, *Apodemus flavicollis* (Mammalia, Rodentia). Genetika. 2015; 47(1):311–321.

[75] Duke Becker SE, Thomas R, Trifonov VA, Wayne RK, Graphodatsky AS, Breen M. Anchoring the dog to its relatives reveals new evolutionary breakpoints across 11 species of the Canidae and provides new clues for the role of B chromosomes. Chromosome Res. 2011; 19:685–708. 10.1007/s10577-011-9233-4 21947954

