# Supplementary material for: Exploring Supernumeraries - A New Marker for Screening of B-Chromosomes Presence in the Yellow Necked Mouse Apodemus flavicollis
Source: PLoS One. 2016 Aug 23;11(8):e0160946. doi: 10.1371/journal.pone.0160946 (PMC4994964; doi:10.1371/journal.pone.0160946)
Supplement: S1 Fig — Alignments with the highest degree of similarity are shown: a) Query 998 bp 3’-end DNA fragment, Sbjct sequences from databases. 998 bp 3’-end DNA sequence showed similarity with serine/threonine-protein kinase VRK1 isoform a and serine/threonine-protein kinase VRK1 isoform b genomic sequences, Mus musculus strain mixed chromosome 12, alternate assembly Mm_Celera Sequence ID: ref|AC_000034.1| b)Mus musculus vaccinia related kinase 1 (Vrk1), transcript variant X7, mRNA, with 98% identities; c) 991 bp 5’-end DNA sequence showed 82% similarity with serine/threonine-protein kinase VRK1 isoform a and serine/threonine-protein kinase VRK1 isoform b genomic sequences, Mus musculus strain mixed chromosome 12, alternate assembly Mm_Celera Sequence ID: ref|AC_000034.1|. (DOC) [file pone.0160946.s001.doc]

a)

Mus musculus strain mixed chromosome 12, alternate assembly Mm_Celera

Sequence ID: [ref|AC_000034.1|](http://www.ncbi.nlm.nih.gov/nucleotide/83274087?report=genbank&log$=nuclalign&blast_rank=12&RID=HMYJ5EEU016) Length: 116539466 Number of Matches: 2

Range 1: 107278765 to 107279395

|  | | | | |
| --- | --- | --- | --- | --- |
| **Score** | **Expect** | **Identities** | **Gaps** | **Strand** |
| 712 bits(385) | 0.0 | 572/658(87%) | 30/658(4%) | Plus/Minus |

Features:

[serine/threonine-protein kinase VRK1 isoform a](http://www.ncbi.nlm.nih.gov/nucleotide/83274087?report=gbwithparts&from=107272752&to=107311473&RID=HMYJ5EEU016)

[serine/threonine-protein kinase VRK1 isoform b](http://www.ncbi.nlm.nih.gov/nucleotide/83274087?report=gbwithparts&from=107272752&to=107311473&RID=HMYJ5EEU016)

Query 334 AGCATGATGTCACAGGATATAACTATAAAACACGCCCACCTGAGATAAACAGCTGAGACA 393

||| |||||| ||||| ||| | |||||| ||| || ||| || | | ||||||| |||

Sbjct 107279395 AGCGTGATGTTACAGGGTATGAATATAAACCACACCTGCCTAAGGTGAGCAGCTGAAACA 107279336

Query 394 GACAGTCCCACGCCTGCAGACCTCTGTGGTCTCTCAGACACCCTCAAATTGTGCCACTCT 453

|||||||||| || ||||| |||||||| ||||||||||||||||| ||||||||||

Sbjct 107279335 AACAGTCCCAC-CCCGCAGAGCTCTGTGGCCTCTCAGACACCCTCAA---GTGCCACTCT 107279280

Query 454 AGTATGGGGGAGCTGAAGACTCTGAATTCAAGTTACTTAAAACTATAAAGCTACTATGTG 513

|| |||||||||||||||||||||||||||||||||||||||||||| ||||||||||||

Sbjct 107279279 AGCATGGGGGAGCTGAAGACTCTGAATTCAAGTTACTTAAAACTATACAGCTACTATGTG 107279220

Query 514 TCTCATTCAGTATTTATGGAAGATTATTAACAGTGCAGTGCCTTATGGTTTAACTTAGTC 573

||||||| | |||||| | ||||||| ||||| | || || | | | ||||

Sbjct 107279219 TCTCATTAAATATTTA-GAAAGATTAATAACA----A-TG---TA--G--T-A---AGTC 107279177

Query 574 TAGTTATCAAAAAAGATGCTTTACAAGATAAATACCAATTACATTGAGAAAAGAAAAAAT 633

|||| ||||||||||||||| |||||||||||||||||||||||||||||| |||||||

Sbjct 107279176 TAGTCATCAAAAAAGATGCTCTACAAGATAAATACCAATTACATTGAGAAAGAAAAAAAT 107279117

Query 634 GAGTAATATTTTCTTACCACTTTCACAACACAGGGTGCGTCACTGCCAACCGGTTTGGAA 693

||| ||||||||||||||||||||||||||||||| ||||||||||||||||||||||||

Sbjct 107279116 GAGCAATATTTTCTTACCACTTTCACAACACAGGGCGCGTCACTGCCAACCGGTTTGGAA 107279057

Query 694 GAATTTGTGTCCGCTGTTTAAAGAGA-GATATTTACATCAGAACAAGGAGTATTTCCATA 752

|||||||||||||||||||||||||| || ||||||||||||||||||||| || |||||

Sbjct 107279056 GAATTTGTGTCCGCTGTTTAAAGAGAAGACATTTACATCAGAACAAGGAGTGTTGCCATA 107278997

Query 753 GACAGGATATGACAGACCCTTAGACCTACAGTCTGAGACCCTCTGAGTTGTGAATCCCAG 812

||||| |||||||| || ||||| || | | ||| ||||||| || ||||||||||||

Sbjct 107278996 GACAGAATATGACACACACTTAGGCC--C--T-TGA-ACCCTCTAAGGTGTGAATCCCAG 107278943

Query 813 ATTCCAAATGGCATTTCAGATCATCATTATAACACTGCACCTAGTCTAAGACATTTTCCA 872

||||||||||||||||||||||||||||||||||||| |||||||||| ||| ||| ||

Sbjct 107278942 ATTCCAAATGGCATTTCAGATCATCATTATAACACTGTACCTAGTCTACGACTGTTTTCA 107278883

Query 873 TATGTAAGATGTATCTAAAAATTTTAGGTTTCTTCAATCTAATTAGATGTTACAGGAAGT 932

|||||| |||||||||| ||||||||||||||||||| |||| ||||||| ||||||||

Sbjct 107278882 TATGTAGGATGTATCTAGCAATTTTAGGTTTCTTCAATTTAATCAGATGTTTCAGGAAGT 107278823

Query 933 TGTTACACAAAGTTAAAACT-TT-CTTTCATTCAGAAGTCCTCTGGTGTTTGGTCTGC 988

|||||||||||||| ||| || |||||||||| |||||||||||||||| || |||

Sbjct 107278822 TGTTACACAAAGTTGCTACTATTGCTTTCATTCACAAGTCCTCTGGTGTTTAGTATGC 107278765

b)

PREDICTED: Mus musculus vaccinia related kinase 1 (Vrk1), transcript variant X7, mRNA

Sequence ID: [ref|XM_006515816.1|](http://www.ncbi.nlm.nih.gov/nucleotide/568979543?report=genbank&log$=nuclalign&blast_rank=3&RID=HMYJ5EEU016) Length: 4067 Number of Matches: 1 Range 1: 881 to 940

| **Score** | **Expect** | **Identities** | **Gaps** | **Strand** |
| --- | --- | --- | --- | --- |
| 106 bits(57) | 5e-20 | 59/60(98%) | 0/60(0%) | Plus/Minus |

Query 648 TACCACTTTCACAACACAGGGTGCGTCACTGCCAACCGGTTTGGAAGAATTTGTGTCCGC 707

||||||||||||||||||||| ||||||||||||||||||||||||||||||||||||||

Sbjct 940 TACCACTTTCACAACACAGGGCGCGTCACTGCCAACCGGTTTGGAAGAATTTGTGTCCGC 881

c)

Mus musculus strain mixed chromosome 12, alternate assembly Mm_Celera

Sequence ID: [ref|AC_000034.1|](http://www.ncbi.nlm.nih.gov/nucleotide/83274087?report=genbank&log$=nuclalign&blast_rank=1&RID=HN3S9G8Z013) Length: 116539466 Number of Matches: 1

Range 1: 107279848 to 107280180

|  | | | | |
| --- | --- | --- | --- | --- |
| **Score** | **Expect** | **Identities** | **Gaps** | **Strand** |
| 272 bits(147) | 5e-70 | 277/336(82%) | 24/336(7%) | Plus/Minus |

Features:

[serine/threonine-protein kinase VRK1 isoform a](http://www.ncbi.nlm.nih.gov/nucleotide/83274087?report=gbwithparts&from=107272752&to=107311473&RID=HN3S9G8Z013)

[serine/threonine-protein kinase VRK1 isoform b](http://www.ncbi.nlm.nih.gov/nucleotide/83274087?report=gbwithparts&from=107272752&to=107311473&RID=HN3S9G8Z013)

Query 677 ACTTACACATGCCTGTCA-TGTGTTCAAATACTGATTTATTAGCTATAAAGTTTACTGAA 735

|||||||||||||||||| |||||||||| ||| |||||||||||||||||||||||||

Sbjct 107280180 ACTTACACATGCCTGTCACAGTGTTCAAATGCTGGTTTATTAGCTATAAAGTTTACTGAA 107280121

Query 736 AAATTATACTTAGTCAATACCTTTAAAATGCCCACTAAAACAAAGGATAGCAatggctca 795

|||||| ||| ||||||||||||||||||| ||||||||| |||| ||| |||||||

Sbjct 107280120 AAATTACACTAAGTCAATACCTTTAAAATGTTCACTAAAACGAAGGCTAGTGGTGGCTCA 107280061

Query 796 gtagccagagcactggctgctctc-cagagaccctgaatggctccccagcac----atgg 850

|| | |||||||||||||||| | ||| |||| |||||| |||||||| ||||

Sbjct 107280060 GTGGTTCGAGCACTGGCTGCTCTTGCGGAGGACCTGGATGGCTTCCCAGCACCCACATGG 107280001

Query 851 cagcacaca-cc--c-g---c-cg-gcttcaggggacccaatgccctcttctg--tc-tc 898

|||| |||| || | | | | |||||||||||||||||| ||||||||| || ||

Sbjct 107280000 CAGCTCACAACCATCTGTAACTCCAGCTTCAGGGGACCCAATGTCCTCTTCTGCCTCCTC 107279941

Query 899 aatggcagcgccaggcatgcacatgatgca--gacatagatgcaggcaaaacacacaaaa 956

||| ||| | |||||||||||||||| |||| |||||||||||||| |||||||

Sbjct 107279940 AATTGCA-CTTT--GCATGCACATGATGCACAGACAAGGATGCAGGCAAAACCCACAAAA 107279884

Query 957 taaacaatttagaaagaaaAATT-CTGGTCTAAGAT 991

||||||| ||||||||||||| ||||||||||||

Sbjct 107279883 TAAACAACTTAGAAAGAAAAAAAGCTGGTCTAAGAT 107279848
