# Supplementary material for: Exploring Supernumeraries - A New Marker for Screening of B-Chromosomes Presence in the Yellow Necked Mouse Apodemus flavicollis
Source: PLoS One. 2016 Aug 23;11(8):e0160946. doi: 10.1371/journal.pone.0160946 (PMC4994964; doi:10.1371/journal.pone.0160946)
Supplement: S1 Table — (DOCX) [file pone.0160946.s002.docx]

| Sample No | Bs | DD5 321 Ct | Avr.Ct | ΔCt | ΔΔCt | 2^-ΔΔCt | LD 251 Ct | Avr.Ct | ΔCt | ΔΔCt | 2^-ΔΔCt | RpS18 Ct |
| --- | --- | --- | --- | --- | --- | --- | --- | --- | --- | --- | --- | --- |
|  |  |  |  |  |  |  |  |  |  |  |  |  |
| 911a | 0 | 24,01 | 23,71 | 2,28 | 0,00 | 1,00 | 24.30 | 24,49 | 3,05 | 0,00 | 1,00 | 20.67 |
| 911b |  | 23.41 |  |  |  |  | 24.67 |  |  |  |  | 22.20 |
| 397a | 1 | 22.00 | 22,17 | -0,15 | -2,42 | 5,35 | 22.91 | 22,67 | 0,35 | -2,70 | 6,50 | 22.24 |
| 397b |  | 22.34 |  |  |  |  | 22.42 |  |  |  |  | 22.39 |
| 441a | 2 | 19.49 | 19,53 | -2,37 | -4,64 | 24,93 | 19.00 | 19,48 | -2,42 | -5,47 | 44,17 | 21.36 |
| 441b |  | 19.57 |  |  |  |  | 19.96 |  |  |  |  | 22.43 |
| 257a | 3 | 16.44 | 16,41 | -5,35 | -7,63 | 197,40 | 17.89 | 17,95 | -3,82 | -6,86 | 116,16 | 21.78 |
| 257b |  | 16.38 |  |  |  |  | 18.00 |  |  |  |  | 21.74 |
| 470a | 0 | 24.00 | 24,18 | 3,60 | 0,00 | 1,00 | 24.31 | 24,42 | 3,84 | 0,00 | 1,00 | 20,90 |
| 470b |  | 24.36 |  |  |  |  | 24.52 |  |  |  |  | 20.26 |
| 444a | 1 | 21.65 | 21,84 | 0,64 | -2,96 | 7,78 | 21.44 | 21,41 | 0,21 | -3,63 | 12,38 | 21,12 |
| 444b |  | 22,03 |  |  |  |  | 21.38 |  |  |  |  | 21.28 |
| 801a | 2 | 19.31 | 19,44 | -1,68 | -5,28 | 38,85 | 20,10 | 20,49 | -0,63 | -4,47 | 22,16 | 21,02 |
| 801b |  | 19.57 |  |  |  |  | 20,87 |  |  |  |  | 21.22 |
| 1054a | 3 | 17.81 | 18,01 | -2,94 | -6,54 | 93,05 | 19,10 | 18,99 | -1,96 | -5,80 | 55,72 | 20,90 |
| 1054b |  | 18.20 |  |  |  |  | 18,87 |  |  |  |  | 21.00 |
| 462a | 0 | 24.36 | 24,53 | 2,16 | 0,00 | 1,00 | 24.00 | 23,98 | 1,61 | 0,00 | 1,00 | 22.51 |
| 462b |  | 24.70 |  |  |  |  | 23.95 |  |  |  |  | 22.23 |
| 324a | 1 | 22.90 | 22,45 | 1,03 | -1,14 | 2,20 | 22.00 | 21,65 | 0,23 | -1,38 | 2,60 | 21,85 |
| 324b |  | 22.00 |  |  |  |  | 21.30 |  |  |  |  | 21,00 |
| 1101a | 2 | 19.80 | 19,78 | -1,82 | -3,98 | 15,73 | 20.60 | 20,70 | -0,89 | -2,50 | 5,64 | 21,12 |
| 1101b |  | 19.75 |  |  |  |  | 20.80 |  |  |  |  | 22,06 |
| 439a | 3 | 19,06 | 19,13 | -2,59 | -4,75 | 26,82 | 19.39 | 19,63 | -2,09 | -3,69 | 12,91 | 21.48 |
| 439b |  | 19,20 |  |  |  |  | 19.87 |  |  |  |  | 21.95 |
| 1103a | 0 | 23.67 | 23,49 | 2,46 | 0,00 | 1,00 | 23.27 | 23,35 | 2,32 | 0,00 | 1,00 | 21,45 |
| 1103b |  | 23,30 |  |  |  |  | 23.42 |  |  |  |  | 20.60 |
| 859a | 1 | 20.95 | 21,07 | 0,19 | -2,28 | 4,84 | 22.29 | 22,21 | 1,32 | -1,00 | 2,00 | 21.42 |
| 859b |  | 21.19 |  |  |  |  | 22,12 |  |  |  |  | 20.35 |
| 400a | 2 | 19.61 | 19,81 | -1,86 | -4,32 | 19,90 | 20.60 | 20,60 | -1,06 | -3,38 | 10,41 | 22,01 |
| 400b |  | 20.00 |  |  |  |  | 20,60 |  |  |  |  | 21.31 |
| 1159a | 3 | 18,11 | 17,70 | -4,11 | -6,57 | 95,01 | 19.85 | 19,55 | -2,26 | -4,58 | 23,92 | 22,11 |
| 1159b |  | 17.28 |  |  |  |  | 19.24 |  |  |  |  | 21.50 |
